# Evidence of Conjoint Activation of the Anterior Insular and Cingulate Cortices during Effortful Tasks

**DOI:** 10.3389/fnhum.2014.01071

**Published:** 2015-01-26

**Authors:** Maria Engström, Thomas Karlsson, Anne-Marie Landtblom, A. D. (Bud) Craig

**Affiliations:** ^1^Department of Medical and Health Sciences, Linköping University, Linköping, Sweden; ^2^Center for Medical Image Science and Visualization (CMIV), Linköping University, Linköping, Sweden; ^3^Department of Behavioural Sciences and Learning, Linköping University, Linköping, Sweden; ^4^Linnaeus Centre HEAD, Linköping University, Linköping, Sweden; ^5^Department of Clinical and Experimental Medicine, Linköping University and UHL, County Council, Linköping, Sweden; ^6^Department of Clinical and Experimental Medicine, Linköping University, Linköping, Sweden; ^7^Atkinson Research Laboratory, Barrow Neurological Institute, Phoenix, AZ, USA

**Keywords:** functional magnetic resonance imaging, working memory, visual perception, forebrain asymmetry

## Abstract

The ability to perform effortful tasks is a topic that has received considerable interest in the research of higher functions of the human brain. Neuroimaging studies show that the anterior insular and the anterior cingulate cortices are involved in a multitude of cognitive tasks that require mental effort. In this study, we investigated brain responses to effort using cognitive tasks with task-difficulty modulations and functional magnetic resonance imaging (fMRI). We hypothesized that effortful performance involves modulation of activation in the anterior insular and the anterior cingulate cortices, and that the modulation correlates with individual performance levels. Healthy participants performed tasks probing verbal working memory capacity using the reading span task, and visual perception speed using the inspection time task. In the fMRI analysis, we focused on identifying effort-related brain activation. The results showed that working memory and inspection time performances were directly related. The bilateral anterior insular and anterior cingulate cortices showed significantly increased activation during each task with common portions that were active across both tasks. We observed increased brain activation in the right anterior insula and the anterior cingulate cortex in participants with low working memory performance. In line with the reported results, we suggest that activation in the anterior insular and cingulate cortices is consistent with the neural efficiency hypothesis (Neubauer).

## 1 Introduction

The ability to perform effortful tasks is a topic that recently has received considerable interest in the research of higher functions of the human brain (Engström et al., 2013; Kurzban et al., 2013; Vassena et al., 2014; Zekveld et al., 2014). Functional neuroimaging studies have shown conjoint activation in the anterior insular cortex (AIC) and the anterior cingulate cortex (ACC). These studies have shown that the AIC and ACC bilaterally are jointly activated across a wide range of cognitive tasks (Medford and Critchley, 2010; Nelson et al., 2010). Several recent reports thus identified the AIC and the ACC as brain network hubs that guide goal-oriented behavior (Dosenbach et al., 2006; Cole and Schneider, 2007; Sridharan et al., 2008; Wittmann et al., 2010; Veroude et al., 2013). It has been proposed that one important role of the AIC + ACC network is to detect salient events and to regulate higher-order processes by facilitating switching between different brain nodes (Dosenbach et al., 2006; Cole and Schneider, 2007; Sridharan et al., 2008; Craig, 2010; Menon and Uddin, 2010). Since signals from AIC precede signals from ACC, it is hypothesized that the AIC engenders awareness and the ACC engenders volitional action (Craig, 2009). In primates, the AIC and ACC have reciprocal connections in both granular and dysgranular layers (Mesulam and Mufson, 1982; Vogt and Pandya, 1987). It is suggested that the von Economo neurons, which are predominantly located in AIC + ACC in humans and great primates, form the connections between these areas (von Economo, 1926; Allman et al., 2010). A functional imaging study of the visual inspection time task (Deary et al., 2004) inferred that progressively increasing activity in the AIC and ACC bilaterally at progressively decreasing (i.e., more difficult) inspection times reflected an “effort-related” mental process. If the AIC + ACC network is fundamental for effort-related processes, then proportional activity should be observable in a wide variety of challenging tasks, and perhaps could directly relate to individual differences in mental capacity.

The present study directly addresses the hypothesis that the AIC and ACC are important network hubs that are recruited for optimal performance in any effortful task. In keeping with the ideas firstly presented in the seminal contribution of Kahneman (1973), we define effort to denote the effects of task-difficulty modulations, which is to say, externally triggered effort in comparison to internally trigged effort. However, we also acknowledge the fact that “mental effort” also is related to attention and a person’s own endeavors or arousal as described by others (Jansma et al., 2007; Wild et al., 2012). In the present study, we investigated the AIC + ACC hypothesis using challenging, effort-related tasks in two different modalities:

1. Working memory, using the reading span task, which challenges complex, executive working memory.
2. Visual perception, using the inspection time task.

Working memory is a limited capacity system that is sensitive to difficulty or levels of complexity, and prior evidence has shown activation related to mental effort or cognitive demand (Honey et al., 2002; Jansma et al., 2007; Nyberg et al., 2009). Cognitive processes involved in working memory include focused attention and willful action also in the presence of distracting events. Thus, working memory is an important mental capacity that is tightly coupled to everyday functions such as problem solving, reasoning, and communication. It has been shown that measures of working memory are strongly correlated with performance in other complex cognitive tasks, such as language comprehension and intelligence tasks (Fry and Hale, 1996; Conway et al., 2003). The Daneman and Carpenter listening span task (Daneman and Carpenter, 1980) used in the present study represents complex, executive aspects of working memory. We have previously reported that the functional magnetic resonance imaging (fMRI)-adapted version of this task, which is designed to engage working memory at discrete levels of mental effort, elicits activation in both AIC and ACC (Engström et al., 2009, 2013).

The inspection time task is a challenging perception task that requires heightened awareness and focused attention to briefly presented sensory (visual or auditory) stimuli (Deary and Stough, 1996; Grudnik and Kranzler, 2001). Deary et al. (2004) showed that performance in a visual variant of the inspection time task was directly related to activation in the AIC + ACC network.

We hypothesize that working memory performance and visual perception speed both are related in terms of mental effort and contingent on activation in the AIC + ACC network. These two different modalities involve activation in different brain networks (the fronto-parietal network for executive function during working memory execution and the visual network during assessment of inspection time) and thus together provide a test of our hypothesis. We chose to obtain individual performance scores on well-validated behavioral versions of these tasks, and then measure regional hemodynamic responses to neural activity using fMRI while participants performed versions of the same tasks adapted for use in the scanner. Our prediction was that effortful execution in these tasks involves graded activation of AIC and ACC, subject to differences in individual performance. Thus, we predicted that individual scores on the pre-scan, behavioral tasks should be correlated with scores on the scanner versions of these tasks, and that effortful performance of the adapted tasks used in the scanner should produce graded activation in these regions that should correlate both with effort (degree of difficulty) and with individual performance.

## 2 Materials and Methods

### 2.1 Participants

Thirty-two healthy participants (15 males and 17 females) of similar ages (18–20 years, mean = 18.7) were recruited from local high schools for the study. The rational for including participants with this limited age range was to obtain participants with similar educational and cultural background. A licensed nurse performed a clinical interview, and the participants reported no health issues that could influence the investigation. They were explicitly screened for eventual brain surgery, medication, drug or alcohol addiction/abuse, and cognitive impairment. Seven of the participants had vision corrective lenses that were used during the fMRI examination. The other participants had normal visual acuity. All participants were right-handed according to the Edinburgh handedness inventory (Mean: 97, range: 80–100) and they were all native Swedish speakers. All participants gave written informed consent to participate in the study, which was approved by the Regional Ethical Review Board in Linköping.

Image data from one participant were excluded from the working memory component of the fMRI analysis and image data from another participant were excluded from the inspection time component due to scanner artifacts. Yet another participant was excluded from the inspection time image analysis due to inappropriate behavioral responses during this task (≤69% correct answers in all trials). Thus, fMRI data from 31 participants in the working memory task and 30 participants in the inspection time task are reported in this communication.

### 2.2 Procedure

The experiment consisted of two parts. In the first part, the participants were assessed with a battery of behavioral tasks during a period of approximately 90 min. These tasks included measures of intelligence (fluid and verbal intelligence/language), working memory, motor speed, and perceptual speed. The specific tasks that were used in data analysis of this study are described in Section 2.3. After the behavioral assessment, the participants were instructed how to perform the fMRI-adapted versions of these tasks. The second part of the experiment took place in the MR scanner, where four different fMRI tasks were administered: working memory and inspection time, which are reported in the present communication, and a finger tapping and a sentence completion task. The tasks were presented in the same order for each participant: (1) Finger tapping first session, (2) Inspection time first session, (3) Working memory, (4) Inspection time second session, (5) Sentence completion, and (6) Finger tapping second session. The fMRI experiment took approximately 1.5 h. The inspection time task was repeated twice in order to enhance the power of the event-related design. In addition, the participants rated their level of fatigue, depression, anxiety, and sleepiness on a visual analog scale before and immediately after the fMRI experiment.

### 2.3 Behavioral assessment

#### 2.3.1 Working memory

In the listening span task (Daneman and Carpenter, 1980), the participants listened to a set of sentences, half of which were semantically correct and the other half incorrect. Participants were instructed to report whether each sentence was correct or not and to remember the last word in each sentence. This procedure was repeated for groups of two to five sentences; the number of sentences presented as a group provides an ordinal measure of the level of difficulty. After the sentences had been presented, the participants were asked to recall all the target words in correct order. The task was repeated five times at each level of difficulty. This task is described in more detail in our previous study (Engström et al., 2009). The number of correct responses summed over each level of difficulty was recorded as behavioral measure of the working memory task.

#### 2.3.2 Inspection time

**Figure 1:**
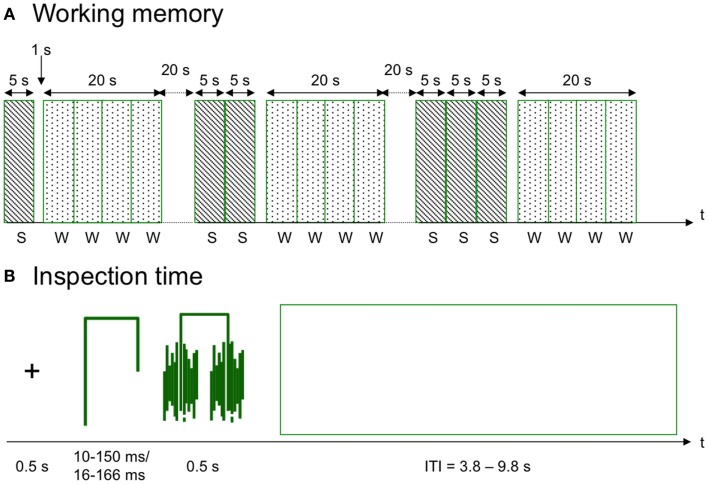
**Schematic diagrams of the tasks**. **(A)** The working memory task consisted of semantically correct and incorrect sentences (S) and words (W) that were either the last word of a sentence or a new word. Each set of 1–4 sentences was repeated five times. **(B)** In the inspection time task, a stylistic figure with either the left or the right leg being longer than the other was showed for a brief interval: 10–150 ms in the behavioral assessment and 117–167 ms in the fMRI version of the task. The figure was preceded by a fixation cross and after presentation the figure was covered by a mask. ITI, intertrial interval.

A computerized version of the inspection time task, as described by Deary et al. (2004), was used in this study. This task examines a participant’s ability to report the asymmetry of a briefly presented visual stimulus: a figure (a stylized reproduction of the Greek letter Π) with either the left or the right leg longer than the other (figure size 68/36 × 36 mm). In the behavioral task of our study, the visual stimuli were presented for durations ranging from 10 to 150 ms, at multiples (modulus) of 10 ms, in pseudo random order (Figure 1). Each stimulus was preceded by the appearance of a fixation cross at the center of the screen during 0.5 s. A mask covering the legs of the figure was presented during 0.5 s following each stimulus. Thereafter a blank screen was shown until a response was provided. After a response, the next stimulus was presented following a random intertrial interval (ITI) of 3.8–9.8 s. The participant was seated 0.5 m from the screen, resulting in an actual viewing angle of approximately 13.5°. The task requirements were described to each participant prior to the examination. It was emphasized that accuracy was more important than response speed. The participants were encouraged and trained to wait a while before responding, however, they had the possibility to answer as soon as the mask appeared on the screen. The task was administered by means of Superlab 4.0 (Cedrus Corporation, San Pedro, CA, USA). Participants responded by pressing one of two pre-defined buttons on the keyboard. The task duration was approximately 20 min. The hit rate at each stimulus duration was recorded as behavioral measure of the inspection time task. A hit rate of 1 corresponds to 100% correct answers. Further, the hit rate at 50 ms was used in the correlation analysis between pre-scanning and fMRI behavioral results, since this stimulus duration was applied in both tasks.

### 2.4 fMRI data acquisition

Image data were acquired on a Philips Achieva (Best, the Netherlands) 1.5 T clinical scanner using the 8-channel sense coil. For fMRI, a blood oxygen level dependent (BOLD) sensitive sequence was used (echo time, TE = 40 ms; repetition time, TR = 3000 ms; flip angle = 90°). Thirty-five transversal slices were acquired in interleaved fashion with 0.5 mm slice gap. The voxel size was 3 mm × 3 mm × 3 mm. The number of acquired volumes (= number of dynamics) for the complex working memory task was 272. The inspection time task was administered in two sessions and the number of dynamics for each session was 271. A 3D sagittal T1W gradient echo sequence was used for structural imaging: TE = 4.6 ms, TR = 25 ms, flip angle = 30°, number of slices = 175, voxel size = 1 mm × 1 mm × 1 mm.

### 2.5 fMRI cognitive tasks

All fMRI tasks were presented to the participants using MR compatible video goggles (Resonance Technology Inc., Northridge, CA, USA) and Superlab software (Cedrus Corporation, San Pedro, CA, USA). The participants made their responses using a Lumi Touch button box (Photon Control Inc., Burnaby, BC, Canada).

#### 2.5.1 Working memory

The working memory task in the scanner was similar to the paper and pencil task described in Section 2.3. A schematic diagram of the task is presented in Figure 1. Each sentence was presented visually for 5 s and the sentences were presented sequentially in groups containing 1–4 sentences, representing four levels of difficulty (Levels 1–4). The participants answered if the sentences were correct or not by pressing one out of two pre-defined buttons on the response pad. After each group of sentences there was a short pause of 1 s, and thereafter four words were presented for 5 s each. The participants were asked to indicate by button presses if these words were target words or new words (lures). At the first level, one word was correct and the remaining three words were lures. At the other levels, two words were targets and two were lures. Five instances of each level of difficulty were presented during the entire task. Each trial (sentences + words) was separated by 20 s ITI. The difficulty levels were presented in the same order for all participants from Level 1 to Level 4. The participants were instructed to answer as fast and as accurately as they could. Task duration was 13.5 min. Working memory scores obtained during fMRI was calculated as hits – false alarms at the most difficult level of the task (Level 4).

#### 2.5.2 Inspection time

The inspection time task in the scanner was similar to the behavioral task described previously (Figure 1). However, due to the slower refresh rate of the MR goggles (60 Hz), longer presentation times were used, ranging from 17 to 167 ms at multiples (modulus) of approximately 17 ms (16.67 ms). The visual size of the stimuli was approximately 8.0°. Stimuli with the shortest presentation times (17, 33, and 50 ms) were replicated 16 times/session, and the remaining stimuli were replicated 8 times/session. The complete set of 104 stimulus presentation events was administered across two different pseudo random variants of 14 min each.

### 2.6 fMRI image analysis

#### 2.6.1 Image preprocessing

All image analysis was performed using SPM8 software (Wellcome Department of Imaging Neuroscience, University College, London, UK) applying the General Linear Model (GLM). Images in each fMRI scan were realigned to correct for movement during scanning and coregistered to the T1W structural image of each participant. The structural images were segmented into gray and white matter images in order to obtain parameter files for normalization to Montreal Neurological Institute (MNI) template. Thereafter, the normalized images were smoothed with 8 mm Gaussian kernel for noise reduction and to ameliorate differences in intersubject localization.

#### 2.6.2 First level analysis

In the first level analysis, contrast images of all participants were calculated. Time derivative and dispersion of the modeled hemodynamic response were included in the analysis as well as the movement parameters from the realignment procedure.

##### 2.6.2.1 Working memory

For the working memory task, we opted to analyze brain activity according to a task-difficulty model. Thus, we analyzed brain activity during word recognition in relation to increasing effort (= increasing difficulty of the administered task) i.e., word recognition after 1, 2, 3, or 4 sentences (Levels 1–4). Sentence reading and word recognition levels 1–4 were modeled as five separate regressors. The onset of the first sentence and the duration of all sentences (one sentence = 5 s, two sentences = 10 s, three sentences = 15 s, four sentences = 20 s) were modeled as the first regressor. The onset of the first word and the duration of the whole word recognition block, duration = 20 s, were modeled as separate regressors for each difficulty level (1–4). Six movement parameters, representing translation in *x*, *y*, and *z* direction and rotation in pitch, roll, and yaw mode, were included as separate regressors in the model. In the analysis, we used a contrast vector of [0 −3 −1 1 3], assuming a linear BOLD response to increasing cognitive demand during word recognition. The first regressor in the design matrix represented the trials when the sentences were presented and this regressor was modeled with a zero weight in the contrast vector. The contrast weights of the four remaining regressors that represented word recognition (Levels 1–4) were chosen such that the sum of the weights was equal to zero. That is to say, the contrast vector [0 −3 −1 1 3] represents [“Sentences” “Word recognition after one sentence” “Word recognition after two sentences” “Word recognition after three sentences” “Word recognition after four sentences”].

##### 2.6.2.2 Inspection time

The inspection time task was also analyzed according to the task-difficulty model. Each stimulus presentation time (17–167 ms) was modeled as a separate regressor. Thus, each stimulus onset was modeled as an event with zero duration, and each event, “17 ms,” “…,” “167 ms,” were clustered together as separate conditions. Six movement parameters, representing translation in *x*, *y*, and *z* direction and rotation in pitch, roll, and yaw mode, were included as separate regressors in the model. In the previous study by Deary et al. (2004), the shortest presentation times, i.e., the most difficult conditions, elicited stronger BOLD responses. Thus in the analysis of the present data, the regressors representing the three shortest presentation times (17, 33, and 50 ms) were contrasted against the remaining covariates. A linear contrast did not significantly change the results. All stimulus presentation times were taken into account, independent of the participant’s individual responses.

#### 2.6.3 Second level analysis

At the second level, we used the contrast images of each participant in one-sample *t*-tests to obtain the pooled activation across all participants for each task separately and in a one-way ANOVA within subject analysis to obtain the conjoint activation across tasks. The conjoint activation was calculated in two ways. Firstly, we calculated the average activation pooled over both tasks. Secondly, we performed a conjunction analysis based on the global null hypothesis. The latter analysis gives information about which brain regions having significant effects of similar direction across both tasks. Images were preliminary thresholded at *p* = 0.001 (uncorrected). After a full-scale significance estimation, results are reported as significant if peak *p*-value *p* < 0.05, corrected for multiple comparisons [family wise error (FWE)]. That is to say, only corrected results are reported.

#### 2.6.4 Regions of interest

Pre-defined image masks of the AIC and ACC were used to restrict the search volume and to make small volume corrections. These pre-defined regions of interest (ROIs) were constructed using the Wake Forest University School of Medicine (WFU) Pickatlas tool (Maldjian et al., 2003). The volumes of the ROIs were: left ACC = 51768 mm^3^, right ACC = 53672 mm^3^, left AIC = 4608 mm^3^, and right AIC = 5152 mm^3^. The WFU Pickatlas was also used to determine anatomical landmarks [defined as gyri/lobes and Brodmann areas (BA)] of brain activation in other regions than those in the pre-defined ROIs.

#### 2.6.5 Contrast estimates

For visual inspection of effort-related brain activation, we calculated the contrast estimates of activation peaks at each difficulty level. Group peaks were obtained from the main difficulty contrast for the working memory and inspection time task, respectively. The contrast estimates representing each regressor in the first level analysis (see above) were calculated for each participant. In AIC, we calculated the contrast estimates in spheres with radius 5 mm centered on the group activation peaks. As activation in ACC was mainly bilateral and the activation peaks in each hemisphere were located close to the midline, we opted to calculate the contrast estimates from midline ACC. Thus, for ACC the mean contrast estimates of the voxels located within an 8 mm × 8 mm × 5 mm box placed on the midline (0 16 46) were calculated. This box covered the most significantly activated ACC subregion.

#### 2.6.6 Correlation analysis

We performed a correlation analysis between working memory performance scores obtained in the pre-scanning part of the experiment and brain activation during the fMRI tasks, using the first level contrast images for each participant. Working memory performance scores were used as a measure of individual capacity in the correlation analysis since working memory was the common denominator of the tasks used in this study. That is, the working memory scores correlated with performance in all tasks (see Results Section 3.1). The working memory scores were entered as covariates in a one-sample *t*-test using SPM8. The resulting images showed brain activation that was positively or negatively correlated with working memory performance.

### 2.7 Statistical analyses

Statistical analyses of behavioral data were performed using Graph Pad Prism 5.0d software (GraphPad Software, Inc., La Jolla, CA, USA) and IBM SPSS Statistics (IBM Corporation, Armonk, NY, USA). The relations between behavioral data acquired before and during fMRI were assessed by Spearman’s rank correlation, since the behavioral responses for the working memory and the inspection time tasks were not Gaussian distributed as assessed by the Kolmogorov–Smirnov normality test. We used one-tailed *t*-tests in the correlation analysis, as we aimed to investigate if behavioral results in this study reproduced previously reported positive correlations between these behavioral measures (Kyllonen and Christal, 1990; Jensen, 1992; Deary and Stough, 1996; Fry and Hale, 1996; Grudnik and Kranzler, 2001; Conway et al., 2003; van Ravenzwaaij et al., 2011).

## 3 Results

### 3.1 Behavioral results

Performance on the working memory and inspection time tasks administered prior to scanning was significantly correlated with performance on the corresponding tasks administered during fMRI (Table 1). There was a lower correlation between performance during the pre-scanning and fMRI versions of the working memory task (*r* = 0.30) compared to the inspection time task (*r* = 0.47). This is explained by the fact that the fMRI version of the task was designed so that all participants should perform well above chance also during the most difficult level of the task. Accordingly, there was a ceiling effect, which counteracts correlation analysis.

**Table 1:** **Correlation between behavioral measures**.

	WM_fMRI_	IT_fMRI_
WM_pre_	0.30*	0.32*
IT_pre_	0.27**	0.47***

The table shows the Spearman correlation coefficient *r* for the correlation between behavioral measures obtained at the pre-scanning session (pre) and the fMRI session (fMRI). Significance levels: *p < 0.05, **p < 0.01, ***p < 0.001. WM, working memory scores; IT, inspection time, correct answers at 50 ms stimulus duration. Working memory scores during scanning were calculated as hits – false alarms at the most difficult level (Level 4).

Working memory performance in the pre-scanning part of the experiment was significantly correlated to the ability to perceive briefly presented visual stimuli in the inspection time task (measured as hit rates at 50 ms stimulus duration), *r* = 0.48, *p* < 0.01.

**Figure 2:**
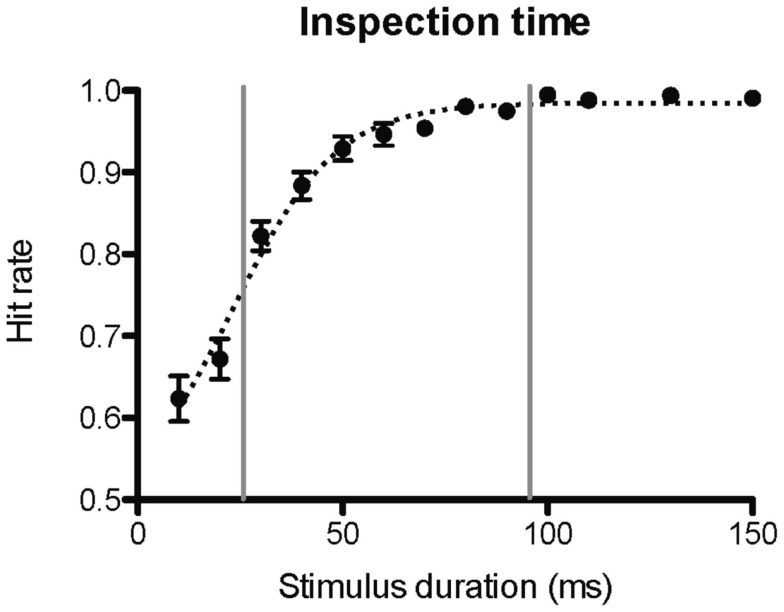
**Inspection time psychometric curve**. The figure shows the psychometric curve of hit rate against stimulus duration for the inspection time task administered prior to the fMRI scanning session. A hit rate of 1 corresponds to 100% correct answers. The vertical lines delimit the range of inspection time durations for which the hit rates correlated with pre-scan working memory performance. The dotted curve shows fitted estimates using a sigmoid function.

The psychometric function of performance on the inspection time task administered prior to scanning plotted against stimulus duration resembles a sigmoid (or, give) function (Figure 2), as expected. The goodness of fit, *r*^2^, of the performance data to a sigmoid curve was 0.70. At the shortest presentation times (10 and 20 ms) the participants answered almost at chance level. At longer presentation times, the response accuracy increased steadily to a perfect or almost perfect hit rate at stimulus durations at or above 80 ms. The hit rates of individual participants for the inspection times between 30 and 90 ms correlated with pre-scan working memory performance (Figure 2). Since different stimulus durations were (by necessity) used for the inspection time tasks performed prior to and during fMRI scanning, the respective psychometric curves could not be directly compared. However, the curves from the pre-scan task and the scanning task have similar shapes, and the hit rates of individual participants were significantly correlated at the only stimulus duration (50 ms) within this window that was used in both trials (Table 1). The mean hit rate at 50 ms of the pre-scanning and the fMRI versions of the inspection time task were 91.6 and 94.9 ms, respectively. There was no significant difference in the hit rate between the two versions of the task, *p* = 0.14. The mean reaction time for all inspection times was 1.4 s. The reaction time of the 17 ms stimulus was significantly longer than the other reaction times, 1.6 ms *p* = 0.013. The remaining reaction times were not dependent on task-difficulty, and were not significantly different from the mean value.

The participants rated higher on both fatigue and sleepiness at the end of the fMRI experiment compared to before (*p* < 0.001). The mean rates of depression and anxiety were lower after the fMRI sessions, however, there were no significant difference in ratings: *p* = 0.35 and *p* = 0.07 for depression and anxiety, respectively.

### 3.2 Brain imaging results

#### 3.2.1 Working memory

##### 3.2.1.1 Brain activation and localization

**Figure 3:**
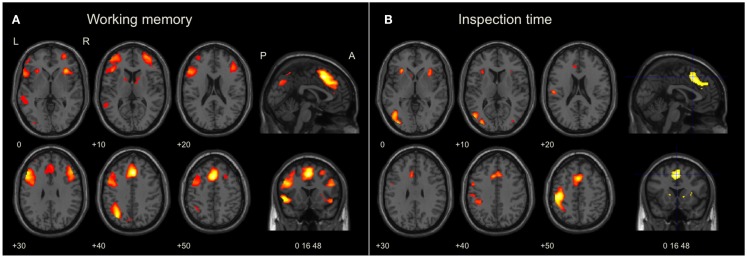
**Brain activation during working memory and visual perception tasks**. The images were significance thresholded at *p* = 0.001, uncorrected, for visualization purpose. Activation in the bilateral AIC and ACC during both tasks **(A,B)** was significant in the ROI analysis (Table 2). **(A)** Brain maps of selected slices showing whole-brain activation during the working memory task in the left fronto-parietal network for executive function, the medial frontal cortex, and in AIC + ACC. **(B)** Brain maps showing whole-brain activation during the inspection time task in the visual and sensory-motor cortices, the medial frontal cortex, and in AIC + ACC. Numbers refer to co-ordinates in Montreal Neurological Institute (MNI) space. L, left; R, right; P, posterior; A, anterior.

As shown in Figure 3A, significant (*p* < 0.05, FWE corrected) activation at the whole-brain level of analysis was found in the AIC + ACC network, as expected: ACC/the medial frontal cortex/BA 32 (4 24 40, *p* < 0.001) and the right AIC (36 24 −4, *p* < 0.001). We also found activation in the fronto-parietal network for executive function related to working memory activity, which was similarly expected: the left middle frontal gyrus (MFG)/BA46 (−48 24 28, *p* < 0.001), the left MFG/BA9 (−50 16 30, *p* < 0.001), the right MFG/BA46 (46 32 24; *p* < 0.001), and the left inferior parietal lobe (IPL)/BA40 (−32 −64 42, *p* < 0.001). In addition, we observed activation in the bilateral fusiform gyrus (−42 −66 −20, *p* = 0.001; 36 −64 −18, *p* = 0.025); an area that is often activated during verbal tasks. All pre-defined ROIs in the AIC + ACC network were significantly activated in both hemispheres (Table 2).

**Table 2:** **Brain activation in regions of interest (ROI)**.

Area	*x*	*y*	*z*	No.	*p*-FWE
**Complex working memory**
Left anterior cingulate cortex (ACC)	−2	16	48	708	<0.001
Right anterior cingulate cortex (ACC)	4	24	40	513	<0.001
Left anterior insular cortex (AIC)	−28	24	−8	259	<0.001
Right anterior insular cortex (AIC)	36	24	−4	204	<0.001
**Inspection time**
Left anterior cingulate cortex (ACC)	−2	16	46	564	<0.001
Right anterior cingulate cortex (ACC)	4	18	46	341	<0.001
Left anterior insular cortex (AIC)	−30	24	4	122	<0.001
Right anterior insular cortex (AIC)	34	18	2	127	<0.001
**Conjunction**
Left anterior cingulate cortex (ACC)	−2	16	46	331	<0.001
Right anterior cingulate cortex (ACC)	4	18	46	248	<0.001
Left anterior insular cortex (AIC)	−28	24	4	91	<0.001
Right anterior insular cortex (AIC)	34	24	4	95	0.001

*The table shows Montreal Neurological Institute (MNI) co-ordinates (*x*, *y*, *z*) of the peak activation in all pre-defined ROIs, number of activated voxels (No.), and family wise error corrected *p*-value at the peak level (*p*-FWE). The working memory and the inspection time task were analyzed according to a task-difficulty model as described in Section 2.6.2*.

##### 3.2.1.2 Brain activation and effort

**Figure 4:**
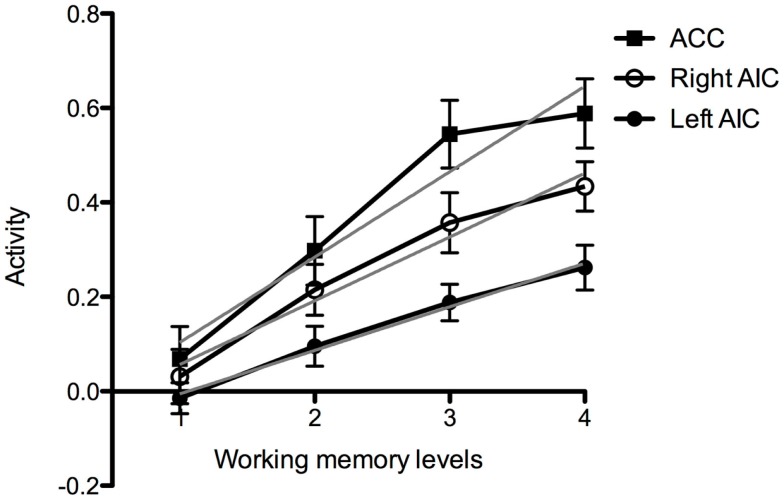
**Working memory activation at increased task-difficulty**. Graph of brain activation in the entire group in the left and the right anterior insular cortex (AIC) and the anterior cingulate cortex (ACC) at the different levels of cognitive demand of the working memory task (Levels 1–4). The *y*-axis refers to activation according to the contrast estimates. The gray line shows fitting according to linear regression.

Visual inspection of activation patterns in bilateral AIC and ACC during the working memory task confirmed a linear increase with increasing cognitive demand (Figure 4). The magnitude of the BOLD response seem to be higher in the right compared to the left AIC. In addition, the group data for activation in the ACC seem to asymptote more quickly (Figure 4).

#### 3.2.2 Inspection time

##### 3.2.2.1 Brain activation and localization

As shown in Figure 3B, clusters with significant peaks (*p* < 0.05, FWE corrected) at the whole-brain level of analysis were found in the AIC + ACC network: ACC/the medial frontal cortex/BA32 (4 18 46; *p* < 0.0001) and the left AIC (−30 24 4; *p* = 0.003). Additional activation was observed in the left sensory-motor cortex with peak activation in the left postcentral gyrus/BA3 (−30 24 4; *p* < 0.0001) and in the visual cortex with peak activation in the left middle occipital gyrus/BA19 (−34 −84 4; *p* = 0.002). All pre-defined ROIs were significantly activated bilaterally (Table 2), and the activation peaks in each ROI were close to the peaks observed in the ROI analysis of the working memory task. The Euclidian distances between the activation peaks in the inspection time and the working memory tasks were in each ROI <10 mm.

##### 3.2.2.2 Brain activation and effort

**Figure 5:**
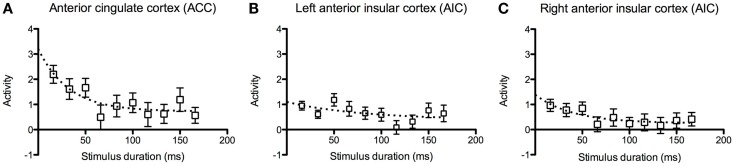
**Inspection time activation at decreased task- difficulty**. Graphs of brain activation in the entire group in **(A)** the left and **(B)** the right anterior insular cortex (AIC) and **(C)** the anterior cingulate cortex (ACC) at the different stimulus durations of the inspection time task. The *y*-axis refers to activation according to the contrast estimates. The plotted points show mean and standard error of the mean (SEM). The dotted curves show fitted estimates using an exponential decay function.

In the ACC, we observed that there was higher activation at shorter stimulus durations and decay toward baseline at longer durations (Figure 5A). The data points for the ACC and the right AIC appeared to fit an exponential decay curve better than a linear model, but the activation in the left AIC was more scattered among the participants (Figures 5B,C).

#### 3.2.3 Conjoint activation

**Figure 6:**
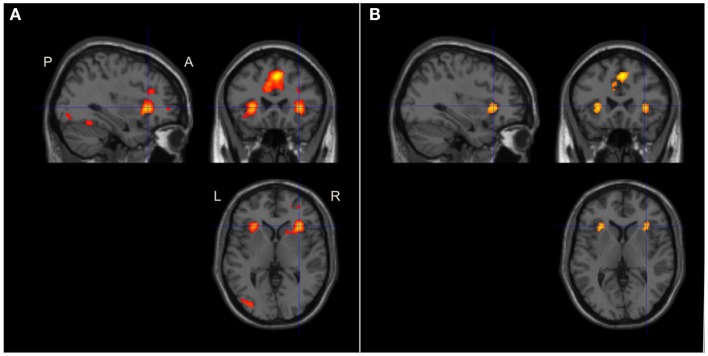
**Conjoint brain activation during the working memory and inspection time tasks The figure shows brain activation in sagittal, coronal, and axial planes**. The images were significance thresholded at *p* = 0.001, uncorrected, for visualization purpose. **(A)** Brain maps showing the global conjoint activation during both tasks. Activation in the AIC + ACC as well as in the left posterior temporal/occipital, posterior parietal, and the left precentral cortices were significant at the whole-brain level of analysis (the latter two areas not shown in the figure). **(B)** Brain maps of the conjunction between the two tasks showing significant whole-brain activation in the ACC and significant small volume corrected activation in bilateral AIC. P, posterior; A, anterior; L, left; R, right.

In the whole-brain analysis of the average effect across both tasks in all participants, we found significant activation, *p* < 0.001, in bilateral ACC (MNI-co-ordinates = [4 18 46]) and AIC (MNI-co-ordinates = [−28 24 2] and [34 24 2]), see Figure 6A. Accordingly, all pre-defined ROIs were significantly activated across tasks. In addition, clusters in the left posterior temporal/occipital cortex, BA 37/19 (MNI-co-ordinates = [−40 −58 −12]; *p* < 0.001), the left IPL, BA 40 (MNI-co-ordinates = [−38 −46 46]; *p* < 0.001), and the left precentral gyrus, BA 6 (MNI-co-ordinates = [−50 2 40]; *p* < 0.001) were activated across tasks at the whole-brain analysis. In the more conservative conjunction whole-brain analysis only the bilateral ACC was significantly activated, *p* < 0.001. However, using a small volume correction in ROIs, significant activation was found in bilateral ACC and AIC also in the conjunction analysis (Table 2; Figure 6B).

### 3.3 Level of performance

**Figure 7:**
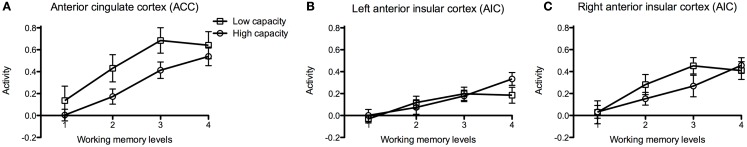
**Brain activation in high and low performance groups during execution of the working memory task**. Graphs of brain activation in participants of the low and high performing groups in **(A)** the anterior cingulate cortex (ACC), **(B)** the left and **(C)** the right anterior insular cortex (AIC) at different levels of cognitive demand of the working memory task (Levels 1–4). The *y*-axis refers to activation according to the contrast estimates. The participants were divided into two groups based on pre-scan working memory performance (median split). The plotted points show mean and standard error of the mean (SEM).

For the working memory task, we extracted the eigen variates within each ROI as measures of brain activity at the different difficulty levels (Levels 1–4). As reported in Section 3.2.1, for the entire group there seemed to be a linear relation between brain activity and task demand in bilateral AIC and ACC. In order to investigate if brain activation during the fMRI task was related to performance on the pre-scan working memory task, we divided the participants into two groups (median split). The activation slopes in ACC appear to be equal in both groups, but the low performing group seems to have higher mean activation level. There was also an apparent reduction in ACC activation in the low performing group at Level 4, which is the most demanding level of this task (Figure 7A). In AIC, the slope and the magnitude seem to be equal in the low and high performing group (Figures 7B,C). Notice, however, which the piecewise linear segments for high and low performers appear to cross on both sides; in the left AIC at Level 3 and in the right AIC at Level 4 (see the Discussion Section 4.2).

**Figure 8:**
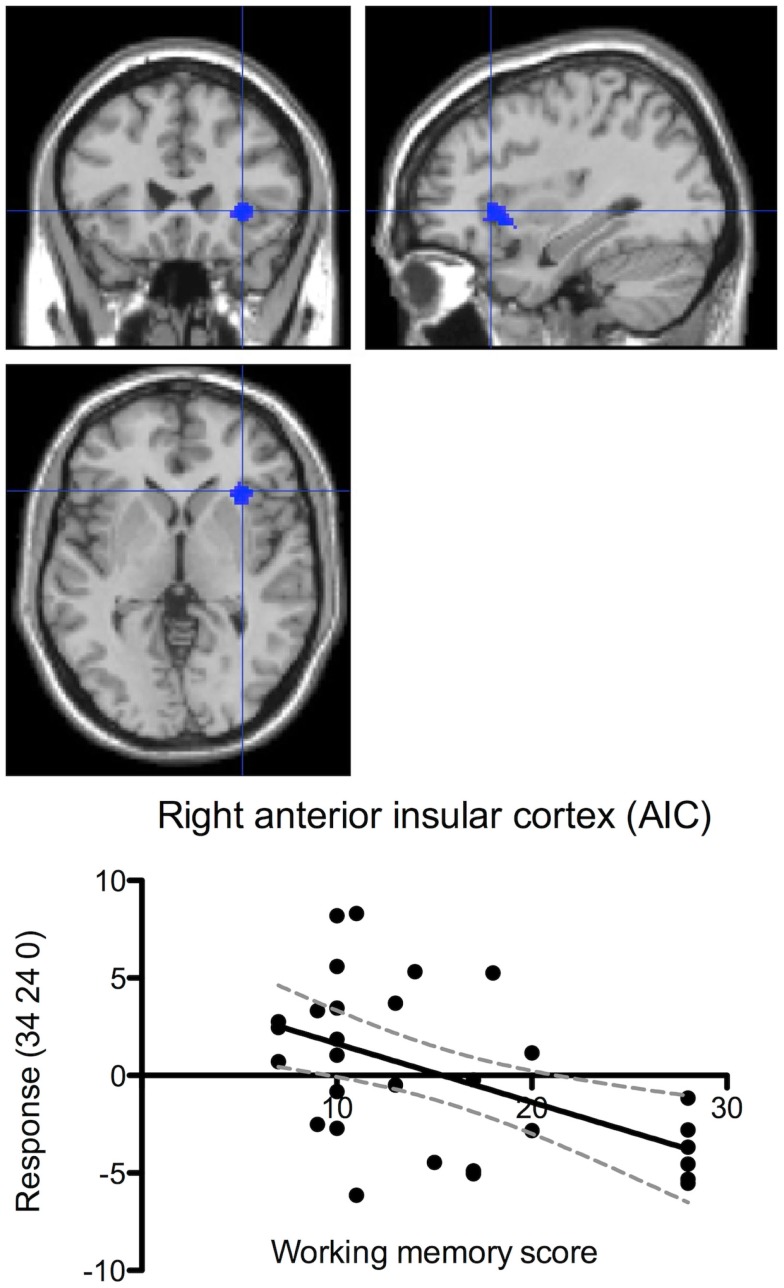
**Performance-correlated brain activation in the right anterior insular cortex during the inspection time task**. The figure shows correlation between individual performance (pre-scan working memory scores) and brain responses (centered around zero) during the inspection time task. Brain activation correlated negatively with performance (corrected *p* = 0.042). The dashed lines indicate the 95% confidence interval. The brain maps show the activation focus in the right AIC at the co-ordinates shown in the ordinate labels.

Data from the most difficult stimulus durations of the inspection time task showed a negative correlation between working memory performance and brain activation in the right AIC, corrected *p* = 0.046 (Figure 8). In other words, increased activation was observed in the right AIC during the most difficult perceptual trials in those individuals with lower scores on the pre-scan working memory task. There was also a tendency of negative correlation between performance and brain activation in the right AIC during the working memory task, but this result did not pass the significance threshold, corrected *p* = 0.072.

## 4 Discussion

Several reviews and meta-analyses have noted that co-activation of the AIC and ACC occurs during a multitude of cognitive and emotional tasks (Craig, 2009; Kurth et al., 2010; Medford and Critchley, 2010; Nelson et al., 2010). In this study, we found that challenging, effort-related tasks in two different modalities (verbal working memory and visual perception) each elicited strong activation in the bilateral AIC and ACC, and further, which average conjoint analysis of the entire group across both tasks also showed activation in the bilateral AIC and ACC. To our knowledge, this is the first time that such conjoint activation has been shown in the same group of participants for which performance on the single tasks also correlates.

### 4.1 Mental effort

The concept of mental effort was re-introduced by Kahneman (1973) who proposed that cognitive processes differ in attentional requirements. In cognitive theory, special emphasis has been placed upon the effortful allocation of cognitive resources (Pribram and McGuinness, 1975, 1992). Although effortful processing is important for our understanding of brain function, only a few neuroimaging studies have investigated the role of cognitive effort in terms of task-difficulty modulations (Jansma et al., 2007; Lim et al., 2010; Chein et al., 2011; Demeter et al., 2011; Wild et al., 2012; Engström et al., 2013). Nevertheless, neuropsychological studies of brain lesion patients have explicitly related the anterior cingulate cortex (ACC) to effortful processing (Mulert et al., 2005; Kohl et al., 2009). Clinical studies have also observed that lack of motivation or “anergia” is associated with focal lesions in the ACC (Damasio and Van Hoesen, 1983; Cohen et al., 1999) and AIC (Ibanez et al., 2010).

In the current study, the working memory and the inspection time tasks were analyzed for the effects of increasing demand. Activation related to task demand during the working memory task was observed in the left MFG, as expected (Cole et al., 2012), however, comparable activation related to task-difficulty was not observed in that area during the inspection time task, similar to prior observations (Deary et al., 2004). We hypothesized that brain activity in AIC and ACC during both the working memory and the inspection time tasks would increase due to increasing task demands, reflecting an effort-related mental process, and that is what we observed. The brain activation during the inspection time task showed a tendency to exponential growth at progressively decreasing stimulus presentation times (= increased task-difficulty), whereas the activation during the working memory task appeared to increase linearly as the task became more difficult.

### 4.2 Individual performance

The pre-scan behavioral assessment showed that working memory scores correlated significantly with performance on the inspection time task. This finding is in line with previous observations that working memory performance is strongly related to mental processing speed (Fry and Hale, 1996). Because performance on the pre-scanning tasks was significantly correlated with performance on the corresponding (albeit simplified) tasks administered during fMRI, the performance measures obtained in the pre-scanning part of the experiment provided a valid correlate of brain activity during fMRI. Thus, the subsequent analyses of cortical activation used pre-scan working memory performance scores as the proxy for individual mental capacity.

When we examined brain activity in relation to individual performance, we observed that participants with higher performance scores showed lower brain activation in the AIC and the ACC. A finding of lower activation in better performing participants in those brain regions that are used for a particular task has been reported in a variety of studies, and it has been ascribed *neural efficiency* (Haier et al., 1988; Gobel et al., 2011; Prat and Just, 2011). According to the neural efficiency hypothesis, the lower brain activation in better performing individuals is due to greater efficiency in the crucial neural network in those individuals. According to Neubauer and Fink (2009), the key findings in the comparison of brain activation between low and high performance groups are: (1) a significant difference in mean activation levels (higher in the low performance group); and (2) a crossing of the two neural activation–task-difficulty curves at a high level of task difficulty. That is to say, they predict that low performers display high brain activation at low and moderate levels of task-difficulty, but very little additional activation at high levels of task-difficulty, where their performance also lags. For high performers, they predict overall lower levels of neural activation that continue to increase in response to increased task-difficulty, along with their performance. Our results are consistent with this theory. In the ACC and the right AIC, the low performing participants displayed higher mean brain activation levels during the working memory task, while the high performing group showed lower activation levels (Figures 7 and 8).

In line with the neural efficiency hypothesis, we observed a tendency of linear trend deviation and subsequent crossing of the respective activation curves for low and high performing participants at a high level of task-difficulty. This is consistent with the inference that the high performing group had more efficient mental resources available, which were required less at low levels of task-difficulty and which supported a higher level of performance at increased levels of task demand. In contrast, the low performing group seemed to have less efficient mental resources available, which were required more at low levels of task-difficulty and which could not support increased performance at increased levels of task demand. Our observations support the neural efficiency hypothesis, however, more research is required to investigate this issue in more detail.

### 4.3 Effort and performance

Data from the present study suggest a dichotomy between the left and the right AIC, since the right (and not the left) AIC showed effort-related brain activation that correlated negatively with performance. It has been shown that the right AIC is activated during stressful events, such as during painful stimuli (Brooks et al., 2002), expectation of painful stimuli (Larsson et al., 2012), and attention to painful stimuli (Kong et al., 2006), as well as anxiety, depression, or post-traumatic stress (Giesecke et al., 2005; Strigo et al., 2010; Simmons et al., 2011). A reinterpretation of a study on recovery in aphasic patients (Saur et al., 2006) by Geranmayeh et al. (2014), emphasizes the role of the right AIC in cognitive effort. In a previous study by us, we also showed that the right hemispheric AIC had increased brain activation as a function of increased effort during complex working memory performance (Engström et al., 2013).

### 4.4 Additional considerations

There are also alternative descriptions of the function of AIC + ACC reported in the literature. These cortical areas have been directly associated with attentional control (Ghatan et al., 1995; Weissman et al., 2006), error monitoring (Taylor et al., 2007; Klein et al., 2013), temporal uncertainty (Limongi et al., 2013), estimation uncertainty (Keri et al., 2004), and effort-related decisions (Walton et al., 2003, 2006; Croxson et al., 2009; Kurniawan et al., 2013). The ACC, in particular, is suggested to be involved with evaluation and selection of choice alternatives to guide future behavior (Kennerley and Wallis, 2009). Several cognitive models attempt to describe the function of ACC. The predicted response-outcome (PRO) model describes the role of the ACC, and the medial prefrontal cortex, in learning and anticipation of action outcomes (Alexander and Brown, 2011). The expected value of control (EVC) model makes an integrative description of the functional diversity in ACC (Shenhav et al., 2013). However, a detailed account for these alternative descriptions of the roles of AIC and ACC, including the medial prefrontal cortex, is out of the scope of the present work since didactic dissociation of the roles of the AIC and ACC in attention, awareness, and mental effort, if possible, requires additional data and analyses.

### 4.5 Limitations

The aim of this study was to investigate effort-related brain activity in the AIC + ACC network and individual performance during tasks in two different modalities. One limitation of the study is that effort was manipulated differently across the tasks; by increasing the task-difficulty during the working memory task and by decreasing the perceptibility during the inspection time task. The two tasks also had different designs (a parametric block design at the working memory task and a parametric event-related design at the inspection time task, and concomitant different task durations. However, the aim of the present study was not to standardize the tasks within the study, but rather to apply tasks, which are used in standardized protocols, and that have documented ability to separate individuals with different capacity.

One might speculate that the long experimental time of approximately 1.5 h could be problematic since fatigue could be expected to play an important role when examining effort. Indeed, the participants rated higher on both fatigue and sleepiness after compared to before the experiment. Another limitation of the study is that we did not estimate the participants’ motivation to engage in the task. It has been shown that activation in AIC–ACC is modulated by the individuals’ motivation in both rewarding and non-rewarding tasks (Clark et al., 2009; Nishimura et al., 2009).

For the purpose of this study, we extracted measures of brain activation in particular ROIs. This methodology has several major concerns, of which the problem of correct anatomical localization might be the most burdensome. It is well known that there are sizeable inter-individual differences in total brain volume and, more significantly, in the size and structural relations of the AIC with adjacent anatomical subregions (Naidich et al., 2004). In the present study, we used a standard anatomical registration and normalization procedure that is commonly used in fMRI studies. This algorithm uses a few anatomical markers and tries to morph the individual brains into a standard template. The method has recognized limitations in accuracy, and we suspect that alignment difficulties are more likely for a deep structure, like the insular cortex (Yuan et al., 2012). Due to the great inter-individual variability in AIC structure (Naidich et al., 2004), we anticipate that future studies could have greater analytical power if individually delineated anatomical ROIs could be used. Alternatively, modern techniques of multivariate analysis in individual brains [e.g., Björnsdotter et al. (2010)] might be more advantageous.

## 5 Conclusion

We hypothesized that the AIC + ACC network is important for effortful processing, so that effort-related activity in this network is a common denominator for different tasks, such as working memory performance and visual perception speed. Our results were consistent with this notion. Within this network, individuals who performed better on behavioral tasks displayed weaker activation when tasks were easy and showed a continued increase in BOLD signals as task demands increased, supporting the neural efficiency hypothesis.

## Author Contributions

A. D. (Bud) Craig was the principal investigator and contributed to the general conception and design of the study, and interpretation of data. Maria Engström and Thomas Karlsson contributed substantially to data acquisition and analysis. Maria Engström was responsible for drafting the work. All authors contributed to the design of the study and revised it critically for important intellectual content. All authors made the final approval of the version to be published and agreed to be accountable for all aspects of the work in ensuring that questions related to the accuracy or integrity of any part of the work are appropriately investigated and resolved.

## Conflict of Interest Statement

The authors declare that the research was conducted in the absence of any commercial or financial relationships that could be construed as a potential conflict of interest.
